# Refining the risk stratification in advanced‐stage classical Hodgkin lymphoma: A critical analysis of clinical prediction models

**DOI:** 10.1111/bjh.70115

**Published:** 2025-08-22

**Authors:** Oguzhan Koca, Ahmet Emre Eskazan

**Affiliations:** ^1^ Department of Internal Medicine Cerrahpasa Faculty of Medicine, Istanbul University‐Cerrahpasa Istanbul Turkey; ^2^ Department of Immunology and Inflammation Faculty of Medicine, Imperial College London London UK; ^3^ Division of Hematology, Department of Internal Medicine Cerrahpasa Faculty of Medicine, Istanbul University‐Cerrahpasa Istanbul Turkey

**Keywords:** A‐HIPI, Hodgkin lymphoma, IPS, prediction model, prognosis

## Abstract

Classical Hodgkin lymphoma (cHL) is a haematological malignancy with high curability; however, prognosis varies significantly based on clinical and biological factors. To enhance risk stratification, several clinical prediction models have been developed over time, particularly for advanced‐stage cHL. The International Prognostic Score (IPS), introduced in 1998, was the first widely adopted model, later refined in 2012 (updated IPS) and further simplified in 2015 (IPS‐3). Despite their prognostic utility, these models have demonstrated declining predictive performance due to advancements in cHL treatment. In response, the HoLISTIC consortium recently introduced the Advanced‐Stage Hodgkin Lymphoma International Prognostic Index (A‐HIPI) in 2023. Unlike previous models, A‐HIPI incorporates continuous variables, aiming to provide a more precise risk assessment. However, its applicability to older patients remains uncertain, necessitating further validation studies. Additionally, none of the existing models incorporate dynamic treatment response markers such as interim positron emission tomography/computed tomography (PET/CT), which have shown strong prognostic value. This review comprehensively discusses the evolution, strengths and limitations of these prediction models, their clinical implications and the necessity for future refinements integrating dynamic biomarkers and treatment response indicators. The integration of machine learning and multi‐omics approaches could further enhance risk stratification, improve treatment personalization and optimize patient outcomes in cHL.

## Graphical abstract

Comparative overview of three major prognostic models for advanced‐stage classical Hodgkin lymphoma (IPS‐7, IPS‐3 and A‐HIPI), summarizing their key variables, age ranges, validation scope and limitations. The figure also highlights future directions, including incorporation of dynamic biomarkers such as interim PET and the application of machine learning with federated learning approaches. These strategies, alongside robust external validation, are essential to refine risk stratification and guide individualized treatment decisions.

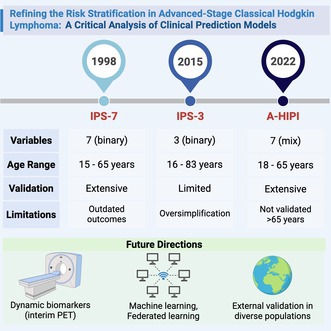

**Abbreviations**
A‐HIPI: Advanced Stage Hodgkin Lymphoma International Prognostic Index
AI: artificial intelligence
AUC: area under the curve
cHL: classical Hodgkin lymphoma
C‐index: Harrell's Concordance Index
EBV: Epstein–Barr virus
EHDS: European Health Data Space
FFP: freedom from progression
FL: federated learning
HL: Hodgkin lymphoma
HoLISTIC: Hodgkin Lymphoma International Study for Individual Care
IPS: International Prognostic Score
ML: machine learning
NHL: non‐Hodgkin lymphoma
OS: overall survival
PFS: progression‐free survival
RT: radiotherapy
TAMs: tumour‐associated macrophages
TRIPOD: Transparent Reporting of a multivariable prediction model for Individual Prognosis or Diagnosis

## INTRODUCTION

Classical Hodgkin lymphoma (cHL) is a highly curable malignancy, yet a subset of patients with advanced‐stage disease continues to experience treatment failure and suboptimal outcomes. In response, multiple risk stratification models have emerged, aiming to improve prognostic accuracy and guide therapeutic decisions, particularly in advanced‐stage cHL.^1^, ^2^, ^3^, ^4^

This review focuses on the evolution, performance and clinical applicability of current prognostic models in advanced‐stage cHL, with particular attention to their validation status, methodological strengths and future directions incorporating personalized risk prediction.

## CURRENT CLINICAL PREDICTION MODELS IN ADVANCED‐STAGE HODGKIN LYMPHOMA

Several prognostic models have been developed for advanced‐stage cHL to estimate long‐term outcomes. The most widely recognized among these are the original International Prognostic Score (IPS, 1998), its updated version (2012), the simplified IPS‐3 model (2015), and more recently, the Advanced‐Stage Hodgkin Lymphoma International Prognostic Index (A‐HIPI, 2023).^1^, ^2^, ^3^, ^4^ Each model differs in its methodological approach and clinical relevance, with varying levels of validation and applicability in the current practice.

The IPS was introduced in 1998 following a pooled analysis of over 5000 patients treated predominantly with doxorubicin‐based regimens. The IPS incorporated seven adverse factors, each contributing modestly to overall survival (OS) and freedom from progression (FFP) prediction.^1^ However, due to evolving treatment strategies and improved outcomes, its discriminatory capacity has declined over time.

An external validation in 2012 confirmed the IPS's retained utility but revealed a narrowing prognostic gap between low‐ and high‐risk groups. In this cohort, only age and haemoglobin level remained independently significant in multivariate analysis.^2^

Subsequently, IPS‐3 was developed in 2015 using data from a phase III North American clinical trial. This simplified model included three predictors (age, stage and haemoglobin) and demonstrated improved OS prediction compared to IPS‐7.^3^ However, its applicability to patients treated with novel or intensified regimens remains uncertain, and it showed limited value for FFP in stage III–IV patients.

While these models have historically guided risk stratification, they show limited relevance in the current clinical practice. The recently developed A‐HIPI model addresses these limitations by incorporating contemporary patient data and retaining continuous variables.^4^ A detailed comparison of model features and performance is provided in Table 1.

**TABLE 1:** Comparative summary of prognostic scoring systems in advanced‐stage classical Hodgkin lymphoma (IPS‐7, updated IPS‐7, IPS‐3).

Original IPS‐7 model
Each factor is assigned 1 point, with a total score ranging from 0 to 7
Serum albumin <4 g/dL
Haemoglobin <10.5 g/dL
Male sex
Age ≥ 45 years
Stage 4 disease
White blood cell count ≥15 000/mm^3^
Lymphocyte count <600/mm^3^ or <8% of total WBC count

Score	5‐year FFP (% ± SD)	5‐year OS (% ± SD)
0	84 ± 4	89 ± 2
1	77 ± 3	90 ± 2
2	67 ± 2	81 ± 2
3	60 ± 3	78 ± 3
4	51 ± 4	61 ± 4
≥5	42 ± 5	56 ± 5

Updated IPS‐7 model (2012)		
Score	5‐year FFP (% ± SD)	5‐year OS (% ± SD)
0	88 ± 5	98 ± 2
1	84 ± 3	97 ± 1
2	80 ± 3	91 ± 2
3	74 ± 3	88 ± 3
4	67 ± 5	85 ± 4
≥5	62 ± 7	67 ± 7

Updated IPS‐7 model, patients under 65 years old		
Score	5‐year FFP (% ± SD)	5‐year OS (% ± SD)
0	88 ± 5	98 ± 2
1	85 ± 3	97 ± 1
2	80 ± 3	92 ± 2
3	74 ± 4	91 ± 3
4	68 ± 6	88 ± 4
≥5	70 ± 8	73 ± 7

IPS‐3 model
Each factor is assigned 1 point, with a total score ranging from 0 to 3
Age ≥ 45 years
Haemoglobin <10.5 g/dL
Stage 4 disease

Score	5‐year FFP (%)	5‐year OS (%)
0	83	95
1	74	85
2	68	75
3	63	52

*Note*: This combined table integrates data from the original and updated IPS‐7 models (including age‐specific results) and the simplified IPS‐3 model. Each model is presented with its respective risk factors and the corresponding 5‐year FFP and OS rates.

Abbreviations: FFP, failure‐free progression; OS, overall survival; SD, standard deviation.

**FIGURE 1:**
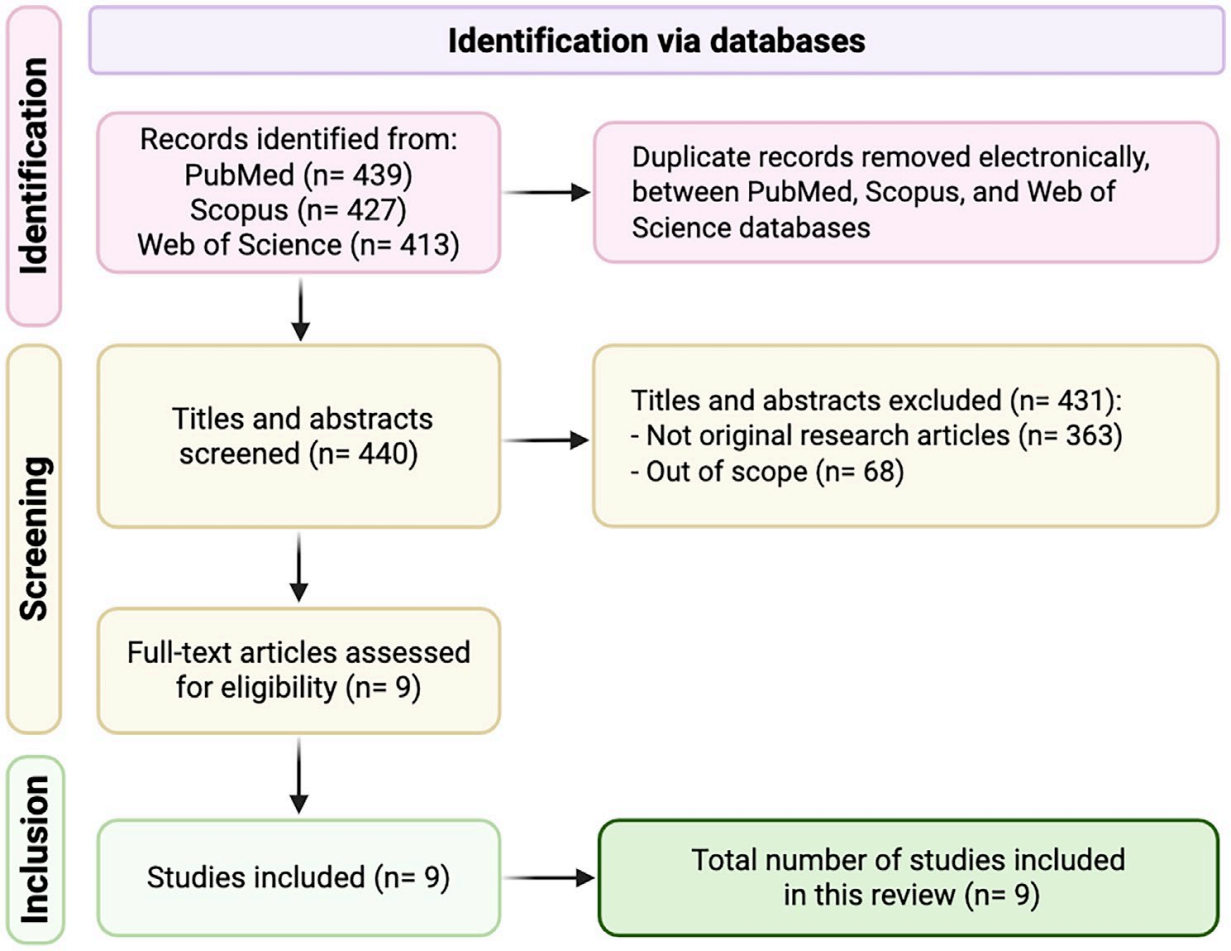
PRISMA‐style flow diagram of the literature search and selection process.

A structured literature search was performed to identify original studies describing or validating clinical prediction models in advanced‐stage cHL. Nine studies met the inclusion criteria and form the basis of this review. The flow of study selection is illustrated in Figure 1.

## ADVANCED‐STAGE HODGKIN LYMPHOMA INTERNATIONAL PROGNOSTIC INDEX

Despite advancements in patient management, primary refractory disease and relapse after salvage therapy continue to be associated with poor prognosis.^5^ Prognostic indices are primarily designed to identify these high‐risk patients who may benefit from more intensive treatment, as well as to recognize low‐risk patients who can be spared unnecessary treatment‐related toxicities. As IPS‐7 and IPS‐3 have become less effective in distinguishing between low‐ and high‐risk patients, new prognostic factors have been investigated. Some of these include tumour‐associated macrophages (TAMs) detected via CD68 positivity,^6^, ^7^, ^8^, ^9^ gene expression profiling from biopsy samples^10^ and EBV DNA expression at diagnosis and during follow‐up.^11^ While these methods have shown promise in predicting prognosis, they have not yet been incorporated into routine clinical practice.

In late 2022, the HoLISTIC consortium developed a new prognostic scoring system called A‐HIPI.^4^ This model was created using data from 4,022 newly diagnosed patients with advanced‐stage (stage IIB, III, IV) cHL enrolled in eight international phase III clinical trials conducted between 1996 and 2014. External validation was performed using data from 1,431 patients with advanced‐stage cHL treated between 1996 and 2019, sourced from four different cancer registries. Patients under 18 or over 65 years old, those with non‐Hodgkin lymphoma histology, those with >50% missing data and those with implausible laboratory values (e.g. albumin <1 g/dL or erythrocyte sedimentation rate <1 mm/h) were excluded from the study. The two primary end‐points were 5‐year progression‐free survival (PFS) and 5‐year OS. As most cHL relapses occur within the first 5 years after diagnosis, this timeframe was chosen for PFS and OS analysis, consistent with previous prognostic models.^4^, ^12^

Potential prognostic factors were assessed, and age, sex, stage, bulky mass, absolute lymphocyte count, haemoglobin and albumin levels were found to be prognostic (Table 2). While sex, bulky disease and haemoglobin were significant only for OS, the remaining four variables were prognostic for both PFS and OS. The definition of bulky disease varied across the eight clinical trials used to develop the model.

**TABLE 2:** Prognostic factors included in the A‐HIPI model.

Variable		5‐year PFS			5‐year OS		
		Beta coefficient	HR	Adjusted Beta coefficient	Beta coefficient	HR	Adjusted Beta coefficient
Age	18–30	−0.026	0.97	−0.024	−0.022	0.98	−0.020
	>30	0.016	1.02	0.014	0.049	1.05	0.046
Female sex					−0.251	0.78	−0.234
Stage	2B						
	3	0.207	1.23	0.184			
	4	0.423	1.53	0.377	0.285	1.33	0.266
Bulky disease					0.312	1.37	
Lymphocyte	100–2000/mm^3^	−0.287	0.75	−0.255	−0.497	0.61	−0.463
	2000–5000/mm^3^	0.188	1.21	0.167	0.396	1.49	0.369
Haemoglobin					−0.124	0.88	−0.116
Albumin		−0.307	0.74	−0.274	−0.406	0.67	−0.379

Abbreviation: A‐HIPI, Advanced‐Stage Hodgkin Lymphoma International Prognostic Index; HR, hazard ratio.

Unlike IPS‐7 and IPS‐3, which categorized risk factors into dichotomous variables (e.g. age ≥45 vs. <45), A‐HIPI utilized continuous data for prognostic factors, reflecting advancements in clinical prediction modelling since IPS‐7 was first introduced in 1998. The A‐HIPI model allows for the input of continuous values for age (18–65 years), albumin (1–6 g/dL), haemoglobin (8–16.5 g/dL) and absolute lymphocyte count (100–5000/mm^3^), while categorical values can be entered for sex, bulky disease and stage. To facilitate its practical use, an online calculator has been developed.^13^

For the entire cohort, 5‐year PFS was 77% (95% CI, 76–78), and 5‐year OS was 92% (95% CI, 91–93). Age and absolute lymphocyte count exhibited non‐linear relationships with PFS and OS. In patients aged 18–30, increasing age correlated with higher PFS and OS, but beyond 30 years, further increases in age were associated with worse PFS and OS. Similarly, lymphocyte counts between 100 and 2000/mm^3^ were associated with improved PFS and OS, but at >2000/mm^3^, further increases in lymphocyte count correlated with poorer PFS and OS.

Following model development, calibration graphs were generated for both A‐HIPI and IPS‐7 in the external validation cohort. A‐HIPI demonstrated good calibration overall, though it slightly overestimated risk in the highest risk decile. In contrast, IPS‐7 overestimated risk across all risk groups.^4^ One limitation of the study was the exclusion of patients over 65 years old, as the cohort was derived from clinical trial participants. Table 3 presents a comparison of patient characteristics from the studies in which these models were developed.

**TABLE 3:** Patient characteristics used in the studies developing clinical prediction models.

	Original IPS‐7		Updated IPS‐7		IPS‐3		A‐HIPI	
	*N*	%	*N*	%	*N*	%	*N*	%
Total	5141		1306		854		5453	
*n* included in model	1618		740		854		4022	
Stage								
Stage 1–2	603	13	308	42	314	37	1106	27.5
Stage 3	2110	45	255	35	321	38	1568	39
Stage 4	1979	42	177	24	208	25	1348	33.5
Histology								
Nodular sclerosis	2936	63	577	78	591	72	2986	74.2
Mixed cellularity	1202	26	53	7	89	11	521	13
Lymphocyte rich	0	0	9	1	14	2	102	2.5
Lymphocyte depleted	124	3	12	2	3	0.4	46	1.1
cHL, NOS	268	6	69	9	105	13	367	9.1
NLPHL	162	3	20	3	16	2	0	0
Other	0	0	0	0	5	0.6	0	0
B symptoms	3274	71	477	64	481	57	2938	73.1
Bulky disease	768	22	288	39	NA	NA	1408	35
Male sex	2882	61	403	54	444	52	2194	54.5
IPS‐7 score								
0	115	7	57	8	78	9	295	7.3
1	360	22	195	26	214	25	944	23.5
2	464	29	195	26	281	33	1281	31.9
3	378	23	155	21	159	19	929	23.1
4	190	12	88	12	74	9	405	10.1
≥5	111	7	50	7	48	6	168	4.2
Age range (median)	15–65 (NA)		16–85 (32)		16–83 (33)		18–65 (33)	
Chemotherapy regimens	Standard doxorubicin‐containing regimens (ABVD); 20% received MOPP or similar		ABVD or ABVD‐equivalent regimens		ABVD or Stanford V		ABVD; Stanford V; MOPPEBVCAD; BEACOPP; escBEACOPP; BEACOPP‐R; COPPEBVCAD; AVD; IGEV; HSCT	
Years of treatment	Before 1992		1980–2010		1996–2006		1996–2014	

*Note*: Adapted from Koca et al.^14^

Abbreviations: FFP, failure‐free progression; HSCT, haematopoietic stem cell transplantation; *n*, number of patients included in the modelling analysis; *N*, total cohort size; NA, not available; NLPHL, nodular lymphocyte‐predominant Hodgkin lymphoma; NOS, not otherwise specified; OS, overall survival.

IPS‐7 and IPS‐3 did not include calibration analyses in their development and validation studies.^1^, ^2^, ^3^ However, A‐HIPI underwent both calibration and discrimination analyses, demonstrating strong predictive performance for mortality and disease progression.^4^ Notably, neither IPS‐7 nor A‐HIPI included patients over 65 years in their original studies, though IPS‐7 was later validated for this age group in its 2012 update.^1^, ^2^, ^4^ Similarly, IPS‐3 was validated for patients over 65 during its development.^3^ In contrast, A‐HIPI has not been validated in patients over 65, limiting its applicability in this population.

A study evaluating A‐HIPI's performance used data from the Danish National Lymphoma Registry, dividing advanced‐stage cHL patients into two groups: those under 65 (*n* = 634) and those over 65 (*n* = 259). The study found that A‐HIPI performed poorly in predicting outcomes for patients over 65.^15^ Consequently, A‐HIPI is currently not recommended for use in patients over 65, and further updates or large‐scale multicentre studies focusing exclusively on older patients may be necessary to develop an age‐specific clinical prediction model.

To enhance the clinical applicability of the A‐HIPI model, a recent study proposed percentile‐based risk groupings due to the model's lack of predefined cut‐offs.^16^ Using the original development and external validation cohorts, the authors stratified patients into low‐, intermediate‐ and high‐risk groups based on A‐HIPI scores and demonstrated acceptable discriminatory performance and calibration.^16^ They also introduced an interactive web‐based calculator to facilitate individualized risk estimation in routine practice, further supporting the model's integration into clinical workflows.^16^

## VALIDATION STUDIES OF THE A‐HIPI MODEL

Due to improvements in prognosis achieved with new chemotherapy regimens for advanced‐stage cHL, the gap between survival predictions from historically established clinical prediction models (IPS‐7, IPS‐3) and actual observed survival outcomes is widening in clinical practice. The A‐HIPI clinical prediction model was developed to bridge this gap; however, validation studies have not yet been completed in regions that differ from the North American, European and Australian populations in which the model was developed such as in terms of socioeconomic status, EBV infection prevalence and cHL subtype distribution.^17^ For clinical prediction models to be applicable to broader populations, they must be validated in different demographic groups. Following the development of the A‐HIPI model, it has been validated in studies conducted in Denmark, Brazil, Italy, Turkey and in a combined Norwegian and Swedish cohort.^14^, ^15^, ^18^, ^19^, ^20^ A comparative summary of the baseline characteristics and outcomes of these validation cohorts is presented in Table 4.

**TABLE 4:** Baseline characteristics of external validation studies of the A‐HIPI model.

Country	Italy^19^	Denmark^15^	Brazil^18^	Norway‐Sweden^20^	Turkey^14^
Year	2023	2023	2024	2024	2025
Cohort size	157	634	694	760	207
Median age	32	N/A	31	37	37
Male (%)	47	N/A	53.7	61.2	58
Stage 3 (%)	24	N/A	27.4	47.4	37.2
Stage 4 (%)	34	N/A	39.5	51.3	34.8
Median follow‐up (months)	57	N/A	60	103	75
5‐year OS (%)	93.7	88	86	91	84.9
5‐year PFS (%)	66.5	76	68.4	87	66.6
C‐index (OS)	0.78	0.74	0.69	0.74	0.74
C‐index (PFS)	0.64	0.63	0.6	0.68	0.61
Calibration intercept (OS)	N/A	−0.06	0.59	0.27	0.03
Calibration slope (OS)	N/A	0.99	0.91	0.88	1.09
Calibration intercept (PFS)	N/A	0.21	0.40	0.89	0.08
Calibration slope (PFS)	N/A	1.18	1.03	1.61	0.99

*Note*: C‐index values refer to model discrimination performance reported in external validation cohorts.

Abbreviations: A‐HIPI, Advanced‐Stage Hodgkin Lymphoma International Prognostic Index; N/A, not available; OS, overall survival; PFS, progression‐free survival.

In validation studies performed in Brazil and Turkey, 5‐year OS and PFS rates were found to be lower than those reported in the literature.^14^, ^18^ Socioeconomic status, an important factor affecting disease outcomes that is not included in clinical prediction models, differs between these countries and the North American and European populations where the models were originally developed.^14^ This difference is thought to contribute to the lower survival rates observed.^14^ These findings highlight the necessity of validating clinical prediction models in regions with different survival outcomes due to variations in histological subtype distribution, socioeconomic factors and EBV infection prevalence.

## ADVANCES IN RISK PREDICTION: SPECIAL CONSIDERATION FOR ELDERLY PATIENTS

Older patients with cHL represent a vulnerable subgroup, often experiencing inferior outcomes due to comorbidities, reduced treatment tolerance and age‐related alterations in immune and tumour biology. Traditional models such as IPS may underestimate risk in this population, as they were primarily derived from younger cohorts receiving intensive therapy. To address this limitation, an elderly‐specific scoring system was recently developed using three parameters: serum albumin level, sex and Eastern Cooperative Oncology Group (ECOG) performance status; named as Hodgkin's Lymphoma Early Death in the Elderly within 12 months (HEDEL12).^21^ This model stratifies patients into low‐, intermediate‐ and high‐risk categories, demonstrating significant discrimination in OS between groups and showing improved prognostic utility over the IPS when applied specifically to older adults with advanced‐stage cHL.^21^

In a separate effort to refine risk assessment in elderly cHL populations, another score was created using age, stage, B symptoms and Charlson Comorbidity Index as key predictors.^22^ This model was validated in a large retrospective cohort of patients aged ≥65 and was shown to independently predict both OS and PFS.^22^ Importantly, these prognostic tools rely solely on routine clinical and laboratory parameters, making them feasible for use in real‐world settings, including centres with limited access to advanced diagnostics. Their incorporation into clinical decision‐making may enable more accurate treatment stratification, balancing efficacy and tolerability in older patients with cHL, an increasingly relevant concern given the ageing global population and expanding therapeutic options.

## INCORPORATING TRIPOD GUIDELINES IN THE EVALUATION OF CLINICAL PREDICTION MODELS

The Transparent Reporting of a multivariable prediction model for Individual Prognosis or Diagnosis (TRIPOD) guidelines provide a structured framework for the development and validation of clinical prediction models.^23^ These guidelines emphasize key methodological aspects, including study design, predictor selection, handling of missing data, internal and external validation and model performance assessment. Applying TRIPOD principles to the IPS‐7, IPS‐3 and A‐HIPI models allows for a critical evaluation of their transparency, generalizability and potential clinical utility.

### Model development and predictor selection

According to TRIPOD, the selection of predictor variables should be clearly justified based on prior evidence and biological plausibility. The IPS‐7 model was developed using retrospective data from a large cohort of patients, identifying seven independent prognostic factors based on their association with OS. However, IPS‐7 does not account for treatment advancements that have improved outcomes over time, potentially limiting its predictive accuracy in contemporary clinical practice.

The IPS‐3 model aimed to simplify risk stratification by reducing the number of variables to three core predictors (age, haemoglobin and disease stage). While this model retains essential prognostic information, the exclusion of other established prognostic factors, such as albumin and bulky disease, raises concerns regarding possible loss of discrimination power.

The A‐HIPI model was designed to enhance prognostic accuracy by incorporating additional variables (e.g. sex, albumin, lymphocyte count, bulky disease) and adjusting for contemporary treatment regimens. This approach aligns with TRIPOD's emphasis on updating models to reflect evolving clinical contexts.

### Handling of missing data

TRIPOD guidelines stress the importance of handling missing data appropriately, either through multiple imputation or sensitivity analyses. The original IPS‐7 model used complete case analysis, which can lead to biased estimates if data are not missing completely at random. For IPS‐3, the handling of missing data is not explicitly reported, making it difficult to assess its robustness in different clinical settings. In contrast, the A‐HIPI model used multiple imputation, a statistically rigorous method that minimizes information loss and improves the reliability of predictions, particularly in heterogeneous patient populations. This methodological improvement strengthens A‐HIPI's adherence to TRIPOD recommendations.

Among the external validation studies, the Brazilian cohort excluded patients with missing or out‐of‐range variables but confirmed in a sensitivity analysis using multiple imputation that results were consistent.^18^ The Turkish cohort also applied multiple imputation for missing predictors.^14^ The combined Norwegian–Swedish cohort used single‐value imputation with regression‐based methods for categorical and continuous variables.^20^ This variability in methodology underscores the importance of adhering to TRIPOD recommendations, as different imputation strategies may influence model performance and generalizability.

### Internal and external validation

Internal validation, typically performed using resampling techniques (e.g. cross‐validation or bootstrapping), is essential for assessing model stability, as recommended by TRIPOD. The IPS‐7 model was validated on the same dataset from which it was derived, which may introduce a risk of overfitting. The IPS‐3 model has been evaluated in independent cohorts, but its external validation remains limited.

The A‐HIPI model included an external validation cohort within the original study, as well as additional validations in diverse geographical populations (Brazil, Denmark, Italy, Turkey and a combined Norwegian–Swedish cohort).^14^, ^15^, ^18^, ^19^, ^20^ This aligns with TRIPOD's emphasis on demonstrating model generalizability across different clinical settings. The inclusion of an external validation sample within the A‐HIPI study itself further enhances its reliability and clinical applicability.

### Performance metrics and calibration

Evaluating prognostic models requires assessing both discrimination (how well a model differentiates between outcomes) and calibration (how accurately predicted risks match observed outcomes).

The IPS‐7 and IPS‐3 models primarily report hazard ratios (HRs) but lack formal reporting of discrimination metrics such as the area under the curve (AUC) or Harrell's Concordance Index (C‐index), which assess a model's ability to correctly rank patients by risk. In contrast, A‐HIPI reports AUC values in both derivation and validation cohorts, demonstrating moderate to strong discrimination (AUC >0.70 in external validations).

Beyond reporting discrimination metrics such as the C‐index, rigorous prognostic model development demands a comprehensive focus on methodological robustness. This includes the avoidance of dichotomizing continuous variables, as cautioned in the methodological literature, which can lead to loss of prognostic information and reduced statistical power.^24^ The A‐HIPI model addresses this limitation by preserving continuous variables and modelling non‐linear associations, in contrast to its predecessors IPS‐7 and IPS‐3.

Moreover, the model's development adheres to TRIPOD guidelines, ensuring transparent reporting, appropriate handling of missing data via multiple imputation and inclusion of both internal and external validation with calibration metrics. Calibration, reflecting how closely predicted risks match actual outcomes, is often underreported in clinical prediction tools, yet it is essential for real‐world applicability. A‐HIPI demonstrated good calibration in the external validation cohort, with only slight overestimation of risk in the highest decile. For PFS prediction, the calibration intercept was −0.40 (95% CI, −0.82 to 0.00) and the slope was 0.83 (95% CI, 0.50–1.16); for OS prediction, the calibration intercept was −0.43 (95% CI, −0.96 to 0.09) and the slope was 0.89 (95% CI, 0.66–1.11).^16^ Independent studies have also supported the model's robust calibration, underscoring its potential clinical utility.

In addition, recent analyses have highlighted that predicted risk distributions derived from the A‐HIPI model are right‐skewed, meaning that most patients are concentrated in the lower risk range.^16^ This skew complicates the task of defining clinically meaningful cut‐off points for high‐ and low‐risk stratification. For example, in the study by Maurer et al., using the 90th percentile of predicted risk as a high‐risk threshold only identified 13% of patients, many of whom still had favourable outcomes.^16^ Such distributions challenge the creation of discrete risk groups and suggest that continuous or percentile‐based approaches may offer greater flexibility. Importantly, this issue underscores the need for prospective validation of risk thresholds before clinical implementation.

These features mark a critical shift towards more rigorous, generalizable and patient‐centred prognostic modelling in advanced‐stage Hodgkin lymphoma. Future refinements should focus on broader external validation, enhanced calibration analyses and implementation of decision curve analysis (DCA) to further improve clinical usability.

### Clinical applicability and implementation

TRIPOD guidelines emphasize the need to assess whether a model is practical for real‐world clinical decision‐making. The IPS‐7 model, though historically significant, may overestimate risk in the modern treatment era, leading to potential overtreatment. IPS‐3 simplifies risk stratification but at the cost of omitting variables that may impact prognosis. The A‐HIPI model is more aligned with current treatment paradigms and has been externally validated, supporting its applicability across different healthcare settings. However, prospective validation in additional populations would further strengthen its credibility.

When evaluating datasets used for model development, substantial variation exists in cohort size and event numbers. The IPS‐7 model was derived from 1618 patients treated prior to the PET era, whereas the A‐HIPI included over 4000 patients, with 858 PFS and 278 OS events. In contrast, the IPS‐3 model did not report the number of events, limiting the assessment of its statistical power. Methodological guidance recommends a minimum of 10–20 outcome events per candidate predictor parameter (events per predictor parameter, EPP) to reduce overfitting and improve model reliability.^25^ Additionally, for time‐to‐event models such as Cox regression, at least 100 outcome events are advised to ensure precise risk estimation, with 200 or more preferred for complex models or external validation.^26^ Notably, a recent Scandinavian registry‐based study on Hodgkin lymphoma (HL) suggested that approximately 200 patients may be sufficient to train models such as A‐HIPI and IPS without substantial loss of predictive performance, providing a pragmatic lower bound for dataset size requirements in this context.^27^ Importantly, the feasibility of meeting these standards has improved over time, as larger, more diverse and well‐annotated datasets have become increasingly available.

Applying TRIPOD guidelines highlights key strengths and limitations of the IPS‐7, IPS‐3 and A‐HIPI models. While IPS‐7 provided a foundational prognostic framework, its applicability has diminished due to treatment advances. IPS‐3 improved simplicity but sacrificed some predictive granularity. A‐HIPI appears to be the most robust and clinically relevant model, given its inclusion of contemporary prognostic factors, external validation and rigorous calibration analyses. Future research should focus on prospective validation, calibration assessment and integration of new biomarkers to enhance the predictive performance of these models in cHL prognosis.

This TRIPOD‐based evaluation underscores the importance of transparent reporting and systematic validation to ensure that clinical prediction models remain relevant and reliable in guiding patient management.

In addition to structured evaluation criteria such as TRIPOD, clinical implementation also requires robust external validation across diverse populations and healthcare settings. Ideally, this includes assessments of both discrimination and calibration in multiple independent cohorts, as well as evaluation of clinical utility through decision‐curve analysis.^28^ Furthermore, prospective or impact studies may provide additional evidence regarding the clinical utility of prediction models, particularly in real‐world settings where performance may differ from controlled cohorts.^29^ Despite promising performance metrics, most models including the A‐HIPI have not yet been tested in such prospective or interventional contexts, which currently limits their adoption in routine care.

This lack of clinical integration is also evident in current international guidelines. Neither the 2018 ESMO Clinical Practice Guidelines, the 2025 NCCN Guideline for cHL nor the 2022 British Society for Haematology Guideline formally incorporate clinical prediction models into their treatment algorithms.^30^, ^31^, ^32^ Instead, current international guidelines predominantly rely on anatomical staging, the presence of B symptoms and PET‐adapted treatment strategies. While these models are widely used for risk stratification in research settings, their role in guiding individualized therapy in routine clinical practice remains undefined. Bridging this gap will require prospective validation, demonstration of clinical utility and integration into guideline‐driven decision‐making pathways.

## FUTURE PERSPECTIVES

Despite significant advancements in the prognosis and treatment of advanced‐stage cHL, there remain several challenges in refining clinical prediction models. Current models, including IPS‐7, IPS‐3 and A‐HIPI, primarily rely on baseline clinical and laboratory parameters. However, the evolving treatment landscape and the increasing use of interim positron emission tomography/computed tomography‐guided therapy suggest that future prognostic models should integrate dynamic factors that reflect real‐time treatment response rather than relying solely on pretreatment characteristics.

One major limitation of existing models is their suboptimal performance in elderly patients. The A‐HIPI model, while demonstrating strong prognostic accuracy in younger populations, has not been validated in patients over 65 years of age, and recent studies indicate that its predictive power is significantly reduced in this group. Future research should focus on age‐specific prognostic models that incorporate not only traditional clinical risk factors but also biological and molecular markers of frailty, immune function and treatment tolerability.

The incorporation of machine learning (ML) and artificial intelligence (AI) algorithms into cHL prognostication represents another promising avenue. AI‐driven models could analyse large‐scale multi‐omics data, including genomic, transcriptomic and radiomic biomarkers, to refine risk stratification and identify novel prognostic factors. Additionally, integrating real‐world data from diverse populations will be critical to ensuring that prediction models remain relevant across different geographic regions and healthcare settings.

### Integration of machine learning and federated approaches

Recent advances in ML have demonstrated considerable potential in healthcare by extracting clinically meaningful patterns from complex datasets such as electronic health records, imaging and laboratory data.^33^ Despite this potential, major challenges remain regarding the generalizability, fairness and interpretability of ML models when applied across heterogeneous healthcare settings. The performance of such models often suffers due to institutional biases, data missingness and variations in data quality and structure. Moreover, privacy regulations and ethical considerations can restrict the central aggregation of sensitive patient data, posing a significant barrier to training robust, generalizable ML‐based prognostic tools.^33^

Federated learning (FL) is a decentralized ML approach that allows multiple institutions to collaboratively develop models without transferring sensitive patient‐level data.^34^ Instead of pooling raw data into a central repository, each institution trains the model locally and transmits only the updated parameters to a central aggregator. These updates are then combined to improve the shared model.^34^, ^35^ This architecture preserves data privacy, complies with local data governance regulations and enables model training across geographically and institutionally diverse datasets. As such, FL has emerged as a viable strategy to address key challenges in multi‐institutional health research, including data fragmentation, privacy concerns and limited generalizability. This makes FL particularly suitable for prognostic modelling in heterogeneous diseases like cHL, where assembling large, diverse datasets across institutions remains operationally and ethically challenging.

This approach is well aligned with emerging initiatives such as the European Health Data Space (EHDS), which aims to connect electronic health records across EU member states and facilitate privacy‐preserving health research and secondary data use.^36^ EHDS could provide the foundational infrastructure needed to support federated learning at scale, allowing the development of next‐generation prognostic models that are more representative, secure and clinically applicable across diverse European populations.

Recent empirical work demonstrated the feasibility of FL for survival prediction in advanced‐stage HL using data from Danish patients, showing that decentralized model training achieved comparable performance to conventional centralized approaches while preserving patient privacy.^37^ These findings provide proof‐of‐concept for the application of FL in HL prognostic modelling and support its potential scalability to broader multi‐national datasets.

Alongside advances in ML, the integration of multi‐omics data including genomics, transcriptomics and proteomics offers new opportunities to enhance risk stratification in cHL. Although evidence in cHL is still limited, early studies suggest that combining digital pathology with genomic profiling may help identify biologically distinct risk groups based on immune cell populations and recurrent genetic alterations.^38^ Similar multi‐omics strategies have been successfully applied in other lymphoma subtypes, such as diffuse large B‐cell lymphoma, supporting their potential utility in cHL.^39^

Finally, future prediction models should aim to go beyond static risk assessment by incorporating adaptive risk stratification, where prognostic scores are updated throughout the treatment course based on individual responses to therapy. This dynamic approach could enable personalized treatment adaptations, minimizing both overtreatment and undertreatment while optimizing patient outcomes.

As the field continues to evolve, future research should focus on developing and validating more robust, personalized and dynamically adaptive prognostic models to further improve survival and quality of life for patients with advanced‐stage cHL.

## CONCLUSION

As treatment modalities and disease outcomes in cHL have evolved over time, previously established clinical prediction models have become less effective in contemporary patient populations. This has necessitated the development of new models that are better calibrated for modern cohorts. In response to these changes, the historical IPS, first introduced by Hasenclever et al. in 1998, was revised in 2012, followed by the introduction of IPS‐3 in 2015. Most recently, with advancements in statistical modelling, the A‐HIPI was developed in 2023 to improve predictive accuracy.

Despite these advancements, there remain significant gaps in prognostic modelling. In particular, IPS models are becoming outdated for older patients, and the A‐HIPI model has not been validated in individuals over 65 years of age, limiting its clinical applicability in this subgroup. Furthermore, current models primarily rely on baseline risk factors at the time of diagnosis, without incorporating dynamic variables that may influence patient outcomes throughout the course of treatment. To further refine risk stratification in cHL, future studies should explore models that integrate treatment selection, interim imaging‐based modifications and other dynamic prognostic factors. The development of such models could enable more personalized and adaptive treatment strategies, ultimately improving long‐term survival and quality of life for patients with advanced‐stage cHL.

## AUTHOR CONTRIBUTIONS

OK collected the related paper and drafted the manuscript. AEE participated in the design of the review and reviewed the manuscript. All authors read and approved the final manuscript.

## CONFLICT OF INTEREST STATEMENT

The authors declare that they have no competing interests.

## Data Availability

The datasets used and/or analysed during the current study are available from the corresponding author on reasonable request.
